# Effect of Teriparatide on Unstable Pertrochanteric Fractures

**DOI:** 10.1155/2015/568390

**Published:** 2015-02-10

**Authors:** Tsan-Wen Huang, Tien-Yu Yang, Kuo-Chin Huang, Kuo-Ti Peng, Mel S. Lee, Robert Wen-Wei Hsu

**Affiliations:** ^1^Division of Joint Reconstruction, Department of Orthopaedic Surgery, Chang Gung Memorial Hospital, 6, West Section, Chia-Pu Road, Putz City, Chia-Yi Hsien 613, Taiwan; ^2^Chang Gung University, 259 Wen-Hwa 1st Road, Kwei-Shan, Tao-Yuan 333, Taiwan; ^3^Division of Orthopaedic Traumatology, Department of Orthopaedic Surgery, Chang Gung Memorial Hospital, 6, West Section, Chia-Pu Road, Putz City, Chia-Yi Hsien 613, Taiwan

## Abstract

We retrospectively analyzed the radiographic and clinical outcomes of unstable pertrochanteric fractures (AO/OTA 31-A2) in 44 patients who underwent dynamic hip screw (DHS) fixation and compared the results with 29 patients who received teriparatide in addition to DHS fixation. A significantly shorter time for fracture healing was recorded in the teriparatide-treated group than in the control group. Rates of lag screw sliding, femoral shortening, and varus collapse were all significantly reduced in the teriparatide-treated group. There were no significant differences with regard to superficial wound infection, pneumonia, urinary tract infection, mortality, malunion, and cutting of the lag screw. The mean overall mobility scores were significantly better in the teriparatide-treated group at 3 and 6 months (*P* < 0.001 and *P* < 0.001, resp.) but not at 12 months or the last follow-up. The pain scores were also significantly better in the teriparatide-treated group at 3 and 6 months (*P* = 0.040 and *P* = 0.041, resp.) but not at 12 months or the last follow-up. Teriparatide improves radiographic outcomes and yields better clinical outcomes at 3 and 6 months postoperatively. The improvement in union time may be important for elderly populations with unstable pertrochanteric fractures to enable them to return to daily activities and reduce morbidity and mortality.

## 1. Introduction

Pertrochanteric fracture is a relatively common and serious clinical issue in geriatric trauma. The injury contributes to high mortality and adverse outcomes in the geriatric population, and the annual number of cases has been estimated worldwide to be as high as 4.6 million by 2025 and 6.26 million by 2050 [1–4]. Osteoporotic hip fracture has a tremendous impact on the health-care system and on society in general. Surgery is usually indicated, but bringing about pain relief, early weight-bearing, and an early return to the preinjury functional level, which is of critical importance to avoiding complications in geriatric patients, remains a challenge for orthopaedic surgeons [3, 4].

Osteosynthesis with an extramedullary or intramedullary device is the standard treatment for these fractures [5, 6], but fixation stability depends on the quality of bone. Osteoporotic bone and fracture comminution are critical for bone anchorage of the implants and subsequent motion between fragments. The poor bone stock decreased pull-out strength of implants and reduced bone regenerative capacity [7]. Despite achieving perfect reduction and optimal positioning of the implant, failure rate in poor bone stock is higher than those in normal bone [8, 9]. The dynamic hip screw (DHS) has been used widely in osteoporotic pertrochanteric fractures. However, lag screw cutting out and excessive sliding with varus and shortening deformity limit its use in such osteoporotic fractures. To avoid such complications, cement augmentation and a trochanter stabilization plate (TSP) have been used. According to the Arbeitsgemeinschaft für Osteosynthesefragen/Orthopaedic Trauma Association (AO/OTA) classification [10], the fractures are classified as either stable (31-A1) or unstable (31-A2 and 31-A3). For unstable pertrochanteric fractures, the orthopaedic community is divided between those using a DHS for these fractures and those who choose a cephalomedullary device. By strictly adhering to surgical principles involving a tip-to-apex distance (TAD) index and quality of reduction, many unstable pertrochanteric fractures can be successfully managed by DHS [11]. However, treatment failure rates for 31-A3 fractures using a DHS are too high to recommend its use. It is acceptable to use a DHS for 31-A2 but generally not for 31-A3 [12].

Even with the improvement in implant design, implant choices, and surgical techniques, pertrochanteric fractures still carry a mortality rate ranging from 2.49% to 33% at one month to one year and constitute a major socioeconomic problem [1, 2]. The orthopaedic surgeon is often the first physician to address these fragility fractures and is in a unique position to make every effort to treat, prevent future fractures, and minimize the need for subsequent revision surgeries [3, 4]. In addition to surgical intervention, many pharmacologic agents have demonstrated antifracture efficacy and may play a valuable role in the treatment of fractures [3, 13, 14]. Among them, the anabolic agent, teriparatide (parathyroid hormone [PTH1-34]), was approved by the US Food and Drug Administration in 2002 and promotes the active building of bone mass by stimulation of proliferation and differentiation of chondrocytes and osteoprogenitor cells [3, 13, 14]. Recent animal studies have demonstrated an acceleration of fracture healing, and the fracture callus in teriparatide-treated animals formed more rapidly, remodeled more quickly, and possessed superior biomechanical properties compared with placebo-treated animals [13–29]. Teriparatide proved to be an attractive agent to enhance fracture healing and limit the risk of nonunion when human trials on fracture healing were performed [13, 14]. Little has been published regarding the role of teriparatide in unstable pertrochanteric fractures [AO/OTA 31-A2] that were managed by DHS [30–36]. This study aimed to assess whether adding teriparatide postoperatively would enhance the success rate of DHS in unstable pertrochanteric fractures using a retrospective analysis of 2 groups of elderly patients with osteoporosis: one that received teriparatide and another that received only calcium replacement therapy.

## 2. Materials and Methods

### 2.1. Demographics

This retrospective study was approved by the Ethics Committee and Institutional Review Board of Chang Gung Memorial Hospital (102-5918B).

Hospital and departmental computer databases were searched for the records of patients who had unstable pertrochanteric fractures (AO/OTA 31-A2) and underwent treatment using a dynamic hip screw (DHS, Synthes, Basel, Switzerland) between January 2009 and January 2012. The different osteoporosis medications, the advantages and disadvantages of osteoporosis treatment based on the guidelines for osteoporosis treatment [37] were explained to the patients and chosen by the patients themselves. The clinical and radiographic data and functional outcomes were reviewed retrospectively.

Inclusion criteria were adult patients older than 65 years who had suffered an unstable pertrochanteric fracture (AO/OTA 31-A2) and received follow-up for a minimum of 24 months. Subjects with an unacceptable reduction of fractures, multiple fractures, pathologic fractures, previous ipsilateral hip or femur surgery, fracture of the opposite hip, developmental abnormality, and use of any antiosteoporotic medications before injury or those with incomplete medical records, radiographic analyses, and functional assessment were excluded. Patients were prescribed teriparatide as suggested by the guidelines for osteoporosis treatment [37]. Teriparatide was subcutaneously administrated with 20 mcg/day for 18 months. Teriparatide was prescribed from the day of surgery. Teriparatide was prescribed before surgery or the durations of teriparatide use less than 18 months were also excluded. The radiographic evaluation, fracture classification, and reduction and fixation quality were reviewed by a blinded observer (Tien-Yu Yang) using digital radiographs on a computer. The medical records and clinical data were reviewed and analyzed by a research associate (Yu-Shuan Lin) who was not part of the operation team and was blinded to group allocation. Patients who met the inclusion criteria and were without exclusion criteria were divided into 2 groups: group A, patients who had DHS fixation and had received calcium replacement therapy; group B, patients that had postoperatively received teriparatide in addition to DHS fixation and calcium replacement therapy.

### 2.2. Radiographic Assessment

All patients had radiographic examinations including an anteroposterior (AP) view of the pelvis and AP and lateral views of the affected hip preoperatively and at 1 day, 2 weeks, and 4 weeks postoperatively and monthly until the healing of the pertrochanteric fracture. The quality of fracture reduction was considered acceptable when the following criteria were met: anatomic or slight valgus alignment on the AP view, alignment with parallel or slight cervical anteversion on the lateral view, and no more than 1 cm of displacement between 2 major fracture fragments [6]. The decreases in neck-shaft angle present on AP view at the last follow-up, when compared with the initial postoperative radiograph, were measured as varus collapse [6]. The magnitude of shortening was measured using the method of Leung et al. [38]. Radiographic union of the fracture was defined as recanalization of the trabeculae or bridging callus visible on both radiograph views; delayed union was defined as no sign of fracture healing after 6 months; nonunion was defined as the absence of bone union after 9 months postoperatively; malunion was defined as femoral shortening of more than 20 mm or varus collapse of more than 15 degrees after comparison with the opposite side. Any change in the position of the implants including lag screw penetration and the amount of lag screw sliding present on the AP view was recorded. Failure of treatment was defined when the following events occurred: (1) penetration of the screw into the hip joint or loosening within the femoral head; (2) breakage of the barrel-plate or its screws; or (3) the patient undergoing a second operation due to implant failure of other causes [6]. All radiographs were reviewed and analyzed by an independent surgeon (Tien-Yu Yang) who was blinded to the group allocation. Bone mineral density (BMD) was measured using the Hologic DXA QDR 4500 (Hologic Inc., Waltham, MA) after surgery on the opposite hip.

### 2.3. Clinical Assessment

Postoperative functional scores were obtained using the mobility score of Parker and Palmer [39]. Hip pain was graded on a 4-point scale: (1) no pain; (2) mild pain not affecting walking or requiring regular analgesic medication; (3) moderate pain affecting walking and/or requiring regular medication; (4) severe pain. Assessment was performed at 3, 6, and 12 months postoperatively and at the last follow-up [6]. The functional scores and pain scores were reviewed and analyzed by a research associate (Yu-Shuan Lin).

### 2.4. Statistical Analysis

All data were entered into an Excel spreadsheet (Microsoft Corp., Redmond, WA) and subsequently copied into SPSS version 13.0 (SPSS Inc., Chicago, IL). Statistical analysis was performed by an independent statistician blinded to the group allocation and surgical outcomes. Student's *t*-test was employed for continuous variables. The level of statistical significance was set at *P* < 0.05. Where appropriate, the *χ* ^2^ test or Fisher's exact test was used for binary variables with the level of statistical significance set at *P* < 0.05.

## 3. Results

There were 211 patients (211 hips) with unstable pertrochanteric fractures (AO/OTA 31-A2) during the enrollment period. Among them, 32 hips treated with an intramedullary device, 11 hips with unacceptable fixation quality, 66 hips with other antiosteoporotic agents, and 21 hips with incomplete data were excluded. This left a total of 81 patients (81 hips) for the final analysis, including 28 men and 53 women with a mean age of 82.3 years (range, 65 to 92 years) at the time of surgery. The mean body height was 156 cm (range, 142 to 177 cm), the mean body weight was 63.8 kg (range, 39 to 95 kg), and the mean body mass index (BMI) was 26.5 kg/m^2^ (range, 19.3 to 38.1 kg/m^2^). The mean follow-up time was 40.1 months (range, 24–60 months). Fifty patients were in group A (patient did not receive teriparatide) and 31 patients were in group B (patient receiving teriparatide). There were no statistical differences in gender, age at time of operation, BMI, ASA classification, and BMD of the contralateral hip between both groups (Table 1).

With regard to surgical and fracture healing complications during the 2-year follow-up, there was no deep infection, delayed union, nonunion, or implant failure in either group. Both groups showed similar findings with regard to superficial wound infection, pneumonia, urinary tract infection, mortality, malunion, and cutting of the lag screw. Three patients in the non-teriparatide-treated group and one patient in the teriparatide group died during the second year of follow-up due to reasons unrelated to the operation. The overall complication rate was not significantly different in the 2 groups (*P* = 0.161) (Table 2).

After excluding the 4 patients who died and 4 patients that required a hip replacement due to cutting out of the lag screw, the functional outcomes of the remaining 73 patients who were successfully treated by DHS fixation were further analyzed at the last follow-up. There were no statistically significant differences with regard to age at time of operation, BMI, BMD, duration of hospital stay, length of follow-up, and tip apex distance (TAD) (Table 3). In addition, there was no significant difference in subsequent fractures between the 2 groups (Table 3). However, the mean union time was longer (14.3 ± 2.8 weeks versus 11.2 ± 1.6 weeks, *P* < 0.001), the sliding of the lag screw was greater (9.6 ± 5.3 mm versus 2.2 ± 1.4 mm,  *P* < 0.001), there was more femoral shortening (13.2 ± 7.4 mm versus 4.2 ± 2.6 mm,  *P* < 0.001), and the varus collapse was more severe (7.7 ± 4.4 degrees versus 2.6 ± 1.7 degrees, *P* < 0.001) in hips without teriparatide treatment compared to hips treated with teriparatide (Table 3). AO-OTA A2 is further subdivided into 3 subgroups and compared. Similar intergroup differences were noted when comparing the union time, sliding of the lag screw, femoral shortening, and varus collapse in the three subgroups (Table 4).

The mean hip pain score was also significantly worse in hips not treated with teriparatide at 3 and 6 months postoperatively (2.5 ± 0.6 versus 2.1 ± 0.7, *P* = 0.040, and 1.8 ± 0.6 versus 1.5 ± 0.5, *P* = 0.041, resp.). The hip pain scores of the 2 groups did not differ after 12 months and at the last follow-up. Statistical significances in mobility scores were achieved with regard to the ability to get about the house (*P* < 0.001 and *P* < 0.001, resp.), the ability to get out of the house (*P* < 0.001 and *P* = 0.002, resp.), the ability to go shopping (*P* < 0.001 and *P* < 0.001, resp.), and the total mobility score (*P* < 0.001 and *P* < 0.001, resp.) at 3 and 6 months postoperatively. The difference in scores between the 2 groups did not achieve statistical significance at 12 months postoperatively and at the last follow-up (Table 5).

## 4. Discussion

The key finding of this study is that teriparatide improves fracture healing, reduction of surgical and fracture healing complications, and better clinical outcome at 3 and 6 months postoperatively in elderly patients with unstable pertrochanteric fracture.

Fragility fractures are frequent injuries affecting patients with osteoporosis and are a burden for the individuals and their family, as well as the health-care system [1, 2]. Pertrochanteric fractures in this population often contribute to pain and immobility and lead to a loss of functioning in daily activities and a loss of quality of life and are associated with high morbidity and mortality [3, 4]. The primary goals in treating pertrochanteric fractures in elderly patients are pain relief, improvement of mobilization, and prevention of complications associated with comorbidities. When performing osteosynthesis for osteoporotic fracture, however, excessive sliding of the lag screw caused by insufficient abutment at the fracture may shorten the femur. Moreover, varus collapse of the proximal fragment and penetration of the screw into the hip joint or loosening within the femoral head may occur due to inadequate screw anchoring in the femoral head [5, 6, 40, 41].

The use of anabolic agents to accelerate fracture healing and enhance bone formation is of interest to orthopaedic surgeons. In a number of studies, recombinant PTH has been demonstrated to have efficacy in treating osteoporosis and reducing subsequent fracture risk, but its benefit in fracture healing remains controversial [3, 13, 14, 35]. Recent studies have found an acceleration of fracture healing with improved biomechanical properties in the fracture callus using recombinant PTH-treated animals [13–29]. Positive effects were also reported for implant fixation, bone-implant contact, and callus distraction osteogenesis in a rat model [19]. Recombinant PTH may prove to be an attractive agent to enhance fracture healing and limit the risk of nonunion when human trials on fracture healing are performed [13, 14].

The beneficial effects of recombinant PTH on fracture healing have been demonstrated recently in case reports [30–33] and prospective randomized controlled trials [34–36]. Aspenberg et al. [34, 35] prospectively studied 120 postmenopausal women and reported an acceleration of fracture healing of the distal radius with the use of PTH1-34. Peichl et al. [36] reported on a prospective randomized controlled study of 65 postmenopausal women with unilateral pelvic fracture and concluded that PTH1-84 accelerates healing in pelvic fractures and improves functional outcome. Animal and clinical data suggest that the main clinical benefits of using recombinant PTH are acceleration of fracture healing and enhancement of bone formation [13, 14, 36]. Although the clinical effects of 2 different recombinant PTHs on fracture healing in postmenopausal women were, respectively, demonstrated, the authors are not aware of any comparative studies of the anabolic effects of the 2 drugs. In this study, we retrospectively analyzed the effect of teriparatide [PTH1-34] on fracture healing in patients with unstable osteoporotic pertrochanteric fractures and found that it significantly reduced the time of fracture healing compared with that in a control group of patients given calcium replacement therapy only.

In treating unstable pertrochanteric fractures (AO/OTA 31-A2), the orthopaedic community is divided between those using a cephalomedullary device for these fractures and those who choose a DHS. The cephalomedullary device is the presence of the nail in the medulla that prevents excessive medialization of the shaft when there is fracture comminution or lack of lateral support for the medial fragment. DHS carries a higher risk of fixation failure because of the inferior biomechanical properties associated with a longer level arm acting on this extramedullary implant. However, many unstable pertrochanteric fractures can be successfully managed by DHS by strictly adhering to surgical principles involving a tip-to-apex distance (TAD) index and quality of reduction [11, 12, 40, 41].

Treatment success for pertrochanteric fractures is a race between fracture healing and implant failure. DHS, an extramedullary fixation device, sustains higher stress compared with the intramedullary device. This extramedullary fixation device also sustains most of the mechanical loads before the fracture fragments abut into a stable position by the sliding of the lag screw in the barrel or until the fracture heals. However, many osteoporotic pertrochanteric fractures treated with DHS fail with implant cutting out or loss of fixation. In this study, implant cutting out occurred in four hips. Three occurred in group A and the remaining one occurred in group B (*P* = 0.504). Of the 3 patients in group A, two were treated with bipolar hemiarthroplasty. In the third patient, the acetabulum was eroded by the hip screw and received a total hip arthroplasty. The patient in group B had lag screw cut out of the femoral head after falling down 2 weeks after surgery and underwent bipolar hemiarthroplasty. The adding of a pharmacologic agent, teriparatide, helps with callus formation, enhances the purchase of the screws, and speeds up bone union in the race between implant failure and fracture healing [34–36]. We found that union time could be reduced by 22% in patients treated with teriparatide. The improvement in union time may be important for elderly populations with unstable pertrochanteric fractures, allowing them to return to daily activities sooner and reducing morbidity and mortality. The current study found improvements in clinical outcomes at 3 and 6 months postoperatively. With regard to complications, there was less morbidity and mortality in the teriparatide-treated group, but no statistical significance was detected between the 2 groups.

Several limitations in this study must be acknowledged. First, this was a retrospective study with all the inherent weaknesses and biases of such study designs. Second, the number of patients was small. In this investigation, we focused on AO/OTA 31-A2 treatment with a DHS and excluded the patient receiving any other pharmacological agent for osteoporosis before injury; the strict inclusion criteria for this investigation were designed to limit the variables in the study, but the strict inclusion criteria also reduced the numbers of subjects and limited the power of the study to detect a clinically significant difference. Further prospective randomized large-scale cohort studies are warranted. Third, all hips reported in this study were treated by DHS, and any hips treated by intramedullary nail were excluded. Some surgeons prefer to treat unstable pertrochanteric fractures using a cephalomedullary device. We do not know whether the adding of teriparatide could also benefit those hips treated with intramedullary nails. Also, this study excluded those hips with an unacceptable reduction of the fracture. We do not know whether teriparatide could benefit those excluded hips. Finally, the current study was limited to AO/OTA 31-A2 pertrochanteric fractures that underwent treatment using a DHS. We had no instances of AO/OTA 31-A1 and AO/OTA 31-A3 pertrochanteric fracture; thus we are unable to comment on whether recombinant PTH would have a similar advantage in treating such conditions.

In conclusion, the present study showed improvement with regard to radiographic fracture healing and reduction of surgical and fracture healing complications in elderly patients with unstable pertrochanteric fracture who were treated with a DHS and teriparatide, compared to treatment with a DHS alone. This study also demonstrated better mobility in the patients who received teriparatide in addition to surgical fixation with a DHS. The improvement in union time and better clinical outcome at 3 and 6 months postoperatively may be important for an elderly population with unstable pertrochanteric fractures, allowing them to return to daily activities sooner, reducing morbidity and mortality.

## Figures and Tables

**Table 1:** Demographic data of the control and teriparatide group patients.

Parameters	Group A	Group B	*P* value
	*N* = 50	*N* = 31	
*Demographic data *			
Gender			0.122
Male	24 (48%)	10 (32.3%)	
Female	26 (52%)	21 (67.7%)	
Age at time of operation (yrs)	81.0 ± 8.4	82.3 ± 9.5	0.188
Body height (cm)	157.3 ± 8.5	152.0 ± 7.0	0.004^*^
Body weight (Kg)	55.2 ± 10.0	54.1 ± 10.0	0.628
Body mass index (kg/m^2^)	22.3 ± 3.5	23.4 ± 3.9	0.201
ASA classification			0.566
ASA I	—	—	—
ASA II	31 (62%)	19 (61.3%)	
ASA III	19 (38%)	12 (38.7%)	
BMD of contralateral hip	−4.3 ± 1.1	−4.4 ± 1.2	0.796
(*T*-score)			

Group A: patients without supplementary pharmacologic treatment.

Group B: patients treated with teriparatide.

Values are shown as mean (standard deviation) or given as the *n* (%).

*P* values for between-group comparisons were determined by the chi-squared test and Fisher's exact test for nominal variables and Student's *t*-test for parametric variables.

^*^Statistically significant (*P* value < 0.05).

**Table 2:** Postoperative complications of patients in the control and teriparatide groups.

Parameters	Group A	Group B	*P* value
	*N* = 50	*N* = 31	
*Variables *			
Postoperative complication			
Superficial wound infection	3 (6.0%)	1 (3.2%)	0.504
Deep wound infection	0	0	—
Pneumonia	3 (6.0%)	1 (3.2%)	0.504
Urinary tract infection	4 (8.0%)	2 (6.5%)	0.581
Mortality	3 (6.0%)	1 (3.2%)	0.504
Delayed union	0	0	—
Nonunion	0	0	—
Malunion	7 (14.0%)	4 (12.9%)	0.584
Cutting of the lag screw	3 (6.0%)	1 (3.2%)	0.504
Implant failure	0	0	—
Overall complication	**23 (46.0%)**	**10 (32.3%)**	**0.161**

Group A: patients without supplementary pharmacologic treatment.

Group B: patients treated with teriparatide.

The values are given as the *n* (%).

*P* values for between-group comparisons were determined by the chi-squared test and Fisher's exact test for nominal variables and Student's *t*-test for parametric variables.

Statistically significant (*P* value < 0.05).

**Table 3:** Outcome measures of patients in the control and teriparatide groups at the last follow-up.

Parameters	Group A	Group B	*P* value
	*N* = 44	*N* = 29	
Variables			
Age at time of operation (yrs)	81.0 ± 9.3	82.1 ± 7.6	0.254
Body mass index (kg/m^2^)	21.9 ± 2.5	22.9 ± 3.1	0.117
ASA classification			0.464
ASA I	—	—	—
ASA II	29 (65.9%)	18 (62.1%)	
ASA III	15 (34.1%)	11 (37.9%)	
Hospital stay (day)	8.6 ± 1.6	7.9 ± 1.6	0.067
BMD of contralateral hip (*T*-score)	−4.0 ± 1.2	−3.9 ± 1.0	0.908
Follow-up (months)	37.3 ± 7.4	36.2 ± 7.1	0.650
Subsequent fracture			
Vertebral fracture	7 (15.9%)	3 (10.3%)	0.421
Hip fracture	2 (4.5%)	1 (3.4%)	0.677
Wrist fracture	2 (4.5%)	2 (6.9%)	0.494
Overall subsequent fracture	**11 (25%)**	**5 (17.2%)**	**0.350**
Union time (weeks)	14.3 ± 2.8	11.2 ± 1.6	<0.001^*^
Tip apex distance (mm)	19.1 ± 2.6	18.8 ± 2.4	0.643
Sliding of lag screw (mm)	9.6 ± 5.3	2.2 ± 1.4	<0.001^*^
Femoral shortening (mm)	13.2 ± 7.4	4.2 ± 2.6	<0.001^*^
Varus collapse (degrees)	7.7 ± 4.4	2.6 ± 1.7	<0.001^*^

Group A: patient without any supplementation of pharmacologic treatment.

Group B: patient treated with teriparatide.

Values are shown as mean (standard deviation) or as the *n* (%).

*P* values for between-group comparison were determined by the chi-squared test and Fisher's exact test which were used for nominal variables.

Student's *t*-test was used for parametric variables.

^*^Statistically significant (*P* value < 0.05).

**Table 4:** Outcome measures of patients in the control and teriparatide subgroups at the last follow-up.

Parameters	AO-OTA A2.1		AO-OTA A2.2		AO-OTA A2.3	
	Group A	Group B	Group A	Group B	Group A	Group B
	*N* = 9	*N* = 6	*N* = 32	*N* = 18	*N* = 3	*N* = 5
Union time (weeks)	14.1 ± 2.3	10.5 ± 2.4^*^	15.3 ± 2.0	11.8 ± 2.7^*^	14.6 ± 2.1	11.4 ± 2.0^*^
Tip apex distance (mm)	18.8 ± 2.6	18.6 ± 2.6	18.7 ± 2.5	18.3 ± 2.0	19.1 ± 2.4	18.8 ± 2.1
Sliding of lag screw (mm)	7.8 ± 3.2	3.2 ± 1.9^*^	9.9 ± 4.9	4.8 ± 2.7^*^	10.1 ± 6.2	4.4 ± 3.7^*^
Femoral shortening (mm)	9.1 ± 4.8	4.6 ± 3.1^*^	13.6 ± 8.6	5.2 ± 2.9^*^	16.1 ± 8.7	7.2 ± 5.2^*^
Varus collapse (degrees)	5.9 ± 3.1	2.4 ± 1.3^*^	7.6 ± 4.7	3.7 ± 2.6^*^	8.7 ± 6.1	2.6 ± 1.6^*^

Group A: patient without any supplementation of pharmacologic treatment.

Group B: patient treated with teriparatide.

Values are shown as mean ± standard deviation.

*P* values for between-group comparisons were determined by Student's *t*-test which was used for parametric variables.

^*^Statistically significant (*P* value < 0.05).

**Table 5:** Clinical outcome measures of patients in the control and teriparatide groups at last follow-up.

	Three months postoperatively		Six months postoperatively		12 months postoperatively		Last follow-up	
Mobility	Group A	Group B	Group A	Group B	Group A	Group B	Group A	Group B
	*N* = 44	*N* = 29	*N* = 44	*N* = 29	*N* = 44	*N* = 29	*N* = 44	*N* = 29
Able to get about the house (0–3)	1.4 ± 0.5	1.8 ± 0.4^*^	2.2 ± 0.4	2.8 ± 0.4^*^	2.8 ± 0.4	2.9 ± 0.2	2.9 ± 0.3	2.9 ± 0.1
Able to get out of the house (0–3)	0.9 ± 0.3	1.3 ± 0.5^*^	1.6 ± 0.5	2.0 ± 0.2^*^	2.1 ± 0.3	2.1 ± 0.3	2.2 ± 0.4	2.3 ± 0.5
Able to go shopping (0–3)	0	1.0 ± 0.4^*^	0.9 ± 0.3	1.4 ± 0.5^*^	1.5 ± 0.5	1.6 ± 0.5	1.6 ± 0.5	1.8 ± 0.5
Total mobility score (0–9)	**2.3 ± 0.7**	**4.1 ± 0.9** ^*^	**4.7 ± 0.8**	**6.1 ± 0.8** ^*^	**6.4 ± 1.0**	**6.8 ± 0.8**	**6.8 ± 1.0**	**7.1 ± 0.9**
Pain score (1–4)	**2.5 ± 0.6**	**2.1 ± 0.7** ^*^	**1.8 ± 0.6**	**1.5 ± 0.5** ^*^	**1.5 ± 0.5**	**1.3 ± 0.5**	**1.2 ± 0.5**	**1.1 ± 0.4**

Group A: patient without any supplementation of pharmacologic treatment.

Group B: patient treated with teriparatide.

Values are shown as mean ± standard deviation.

*P* values for between-group comparisons were determined by Student's *t*-test which was used for parametric variables.

^*^Statistically significant (*P* value < 0.05).
